# Quality of life measurement in women with cervical cancer: implications for Chinese cervical cancer survivors

**DOI:** 10.1186/1477-7525-8-30

**Published:** 2010-03-19

**Authors:** Ying Chun Zeng, Shirley SY Ching, Alice Y Loke

**Affiliations:** 1 School of Nursing, The Hong Kong Polytechnic University, Hong Kong, PR China

## Abstract

**Background:**

Women with cervical cancer now have relatively good 5-year survival rates. Better survival rates have driven the paradigm in cancer care from a medical illness model to a wellness model, which is concerned with the quality of women's lives as well as the length of survival. Thus, the assessment of quality of life among cervical cancer survivors is increasingly paramount for healthcare professionals. The purposes of this review were to describe existing validated quality of life instruments used in cervical cancer survivors, and to reveal the implications of quality of life measurement for Chinese cervical cancer survivors.

**Methods:**

A literature search of five electronic databases was conducted using the terms *cervical/cervix cancer*, *quality of life*, *survivors*, *survivorship*, *measurement*, and *instruments*. Articles published in either English or Chinese from January 2000 to June 2009 were searched. Only those adopting an established quality of life instrument for use in cervical cancer survivors were included.

**Results:**

A total of 11 validated multidimensional quality of life instruments were identified from 41 articles. These instruments could be classified into four categories: generic, cancer-specific, cancer site-specific and cancer survivor-specific instruments. With internal consistency varying from 0.68-0.99, the test-retest reliability ranged from 0.60-0.95 based on the test of the Pearson coefficient. One or more types of validity supported the construct validity. Although all these instruments met the minimum requirements of reliability and validity, the original versions of these instruments were mainly in English.

**Conclusion:**

Selection of an instrument should consider the purpose of investigation, take its psychometric properties into account, and consider the instrument's origin and comprehensiveness. As quality of life can be affected by culture, studies assessing the quality of life of cervical cancer survivors in China or other non-English speaking countries should choose or develop instruments relevant to their own cultural context. There is a need to develop a comprehensive quality of life instrument for Chinese cervical cancer survivors across the whole survivorship, including immediately after diagnosis and for short- (less than 5 years) and long-term (more than 5 years) survivorship.

## Introduction

Cervical cancer is one of the most common types of cancer in developing countries. With nearly 500 000 women developing cervical cancer per year, China's estimated 131 500 new cases constitute 28.8% of the total new cases annually worldwide [1]. Due to widespread screening programs, the majority of cervical cancer cases are being diagnosed in the earlier stages. Along with new and advanced medical treatment, women with cervical cancer have relatively good 5-year survival rates. The overall 5-year survival rate of all stages of cervical cancer among Chinese women has been estimated to be 70.93% [2].

Better survival rates have driven the paradigm in the life-altering burden of cancer care from a medical illness model to a wellness model concerned with the quality of women's lives as well as the length of survival [3]. The current reality of cancer therapies has also led us to recognize the significance of improving the quality of cancer survivors' lives [4]. Quality of life (QOL) is one of the health outcomes that enable healthcare providers to better address the ongoing concerns of cancer survivors.

Due to cultural differences, Chinese cancer survivors may have a different interpretation of QOL. The concept of QOL is defined by Western cancer survivors as being healthy and independent, reclaiming life, psychological well-being or social relationships [5]. Chinese cancer survivors view "normal living", a good working life, happiness, material resources and support from their families as essential indicators of QOL [6,7].

As QOL in cancer survivors varies by treatment, time since diagnosis and cancer sites [8], there is a need to review QOL measurement issues with a focus on specific cancer sites. While Vistad et al. [9] reviewed studies about the impact of cervical cancer on women's QOL, their review revealed little about QOL measurement for this target population. Although Pearce et al. [10] and Zebrack & Cella [11] conducted methodological reviews of QOL measurement in various types of cancer survivors, there is a lack of review articles focusing on QOL measurement in cervical cancer survivors.

### Aims

The purpose of this review was to describe existing validated multidimensional QOL instruments used in cervical cancer survivors, and to reveal implications of QOL measurement for Chinese cervical cancer survivors.

### Framework of quality of life

Quality of life is dynamic and changes over time [12]. Traditional models of QOL are a multidimensional construct of health including physical, psychological, social and spiritual well-being [13]. It has been argued that this traditional framework predominantly focuses on the individual-centered paradigm, and ignores contextual factors that influence QOL [14]. The contextual QOL model proposed by Ashing-Giwa [14] includes both the individual and systemic paradigms, and was adopted as the framework for this review.

Within each level of paradigm, there are four major domains and a variety of components. The individual level consists of (1) General Health domain including components of health status and co-morbidity; (2) Medical Factors domain including components of age at diagnosis and cancer characteristics; (3) Health Efficacy domain including components of health practices, utilization, perceived health efficacy and medical adherence; and (4) Psychological Well-being domain including components of emotional distress, cognitive function, and positive psychological feelings [14]. The systemic level consists of (1) Socio-ecological domain including components of socio-economic status, life burden, social support, and role/relationship changes; (2) Cultural domain including components of spirituality, acculturation, and interconnectedness; (3) Demographic domain including components of chronological age; and (4) Healthcare System domain including components of access to health care and satisfaction with the quality of health care [14].

## Methods

### Searching strategies

Articles published in English or in Chinese from January 2000 to June 2009 were searched for the review. Terms used for searching included *cervical cancer*, *cervix cancer*, *survivors*, *survivorship*, *quality of life*, *measurement, assessment*, and *instruments*, which were searched in five computerized databases: CINAHL, Medline, PsycInfo, Scopus, and the Chinese Journal Full-text Database (CJFD). In this review, the term 'cervical cancer survivor' was adapted to mean a person living with cervical cancer immediately after the initial diagnosis [15].

### The process of search and selections

**Figure 1:**
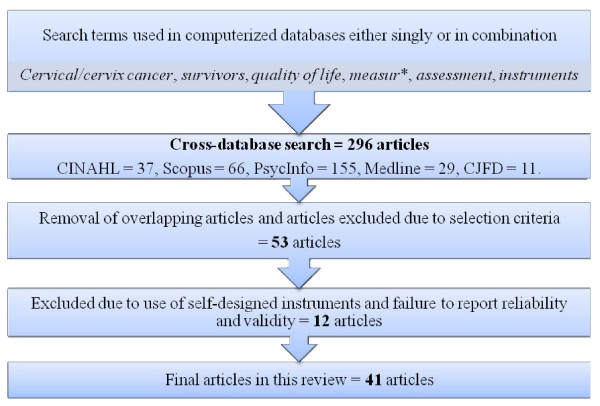
**Flowchart of search and selection process**.

Initially, a total of 296 articles were identified from the literature search of the five databases using the above key words. Duplications of articles and those articles that did not meet the selection criteria were removed. Only 53 articles remained. Twelve of these had used self-designed instruments and did not report reliability and validity. As a result, a total of 41 articles were included. The flowchart of search and selection process was outlined in figure 1.

### Inclusion and exclusion criteria

A checklist was used to select the literature. For inclusion, all of the following criteria had to be fulfilled by the articles: (1) QOL was one of primary outcome measures; (2) women with a diagnosis of cervical cancer constituted the study population; (3) papers were published either in English or in Chinese between January 2000 and June 2009 (at time of search). In terms of exclusion criteria, all qualitative studies, commentaries, editorials, literature reviews, and conference proceedings were excluded from this review. As the concept of QOL is multidimensional (including the physical, psychological, social and spiritual well-being dimensions) [13], studies focusing on a single domain of QOL only were excluded.

### Common types of reliability and validity in QOL measurement

The basic characteristics of a good QOL instrument should demonstrate evidence of adequate reliability and validity [10]. The most common types of reliability reported for QOL questionnaires are internal consistency (assessing the homogeneity of the scale) and test-retest reliability (assessing the stability of the scale) [16]; common types of validity reported by researchers include content validity (to what degree all items in a QOL instrument quantitatively represent the actual content area of the study) and construct validity (how well items reflect the latent variable in question), which can be assessed by convergent/divergent validation, known-group/contrasted-group validation and factor analysis approaches [17].

### The minimum acceptable level of reliability and validity

According to DeVellis [18], the acceptable level of internal consistency by Cronbach's alpha should be above 0.7. Fitzpatrick et al. [19] suggested that instruments examining test-retest reliability within 2-14 days and achieving a Pearson's correlation of over 0.7 were considered to be acceptable. If calculated by the Kappa coefficient or ICC (Intra-class Correlation Coefficient), an item total correlation of at least 0.2 is coded as acceptable [20]. In terms of construct validity, a convergent correlation score above 0.4 is coded as an acceptable standard [21]. By factor analysis, DeVellis [18] suggested that the eigenvalues of factors greater than 0.5 were considered to be acceptable. With known-group validity, the scale can differentiate among the groups [18].

## Results

Among the 41 articles identified, 11 validated multidimensional instruments had been administered to assess QOL among cervical cancer survivors.

### Types of multidimensional QOL instruments

After careful review of the characteristics and use of instruments in these studies, the instruments could be classified into four categories: generic instruments, cancer-specific instruments, cancer site-specific instruments, and survivor-specific instruments.

The generic questionnaires were designed to assess general aspects of QOL. This category included 4 instruments: the 36-item short form of the Medical Outcome Study questionnaire (SF-36) [22,23], the World Health Organization Quality of Life-Brief (WHOQOL-BREF) [24,25], the Quality of Life Index (QLI) [26], and the European Quality of Life Scale-5 dimensions (EQ-5D) [27]. The cancer-specific instruments were designed to assess the QOL of cancer patients as a whole. This category contained 3 instruments: the Cancer Rehabilitation Evaluation System-Short Form (CARES-SF) [28], the European Organization for Research Treatment's Quality of Life Questionnaire (EORTC QLQ-C30) [29,30], and the Functional Assessment of Cancer Therapy-General (FACT-G) [31,32]. The cancer site-specific QOL instruments were developed to measure the QOL of cervical cancer patients. This category consisted of 3 scales: the EORTC Quality of Life Questionnaire-Cervix-24items (QLQ-Cx24) [33], the Functional Assessment of Cancer Therapy-Cervix (FACT-Cx) [34,35], and the Quality of Life Instruments for Cancer Patients-Cervical Cancer (QLICP-CE) [36]. The survivor-specific category included the Cancer Survivors' Unmet Needs (CaSUN) scale [37], which was developed to assess QOL among long-term cancer survivors using a needs-based approach.

A brief description of each instrument, including categories, origin of countries and sample items, is shown in additional file 1. While these instruments varied in length and emphasis, they shared the common perspective that QOL is a multidimensional concept including physical, psychological, social and spiritual well-being, and environmental conditions.

### The paradigms, domains, components and distribution of items

There was a great variation in the domains and number of items in these 11 multidimensional QOL instruments. While these instruments were developed by different researchers and framed by different QOL models with combinations of related domains, it was considered beneficial to identify the common shared domains and components adopted to assess QOL among cervical cancer survivors. The item distribution of these 11 multidimensional instruments was tabulated according to Ashing-Giwa's contextual QOL model [14] (additional file 2).

Additional file 2 shows that, at the individual level, items in these QOL instruments mainly covered the domains of 'general health' and 'psychological health', with few covering 'medical factors' and 'health efficacy'. At the systemic level, these QOL instruments mainly included items to measure the socio-ecological domain, i.e. in the components of 'socio-economic status', 'social support', and 'role/relationship changes'. Very few items in these instruments covered the 'cultural domain' or the 'healthcare system'.

### The psychometric properties of multidimensional QOL instruments

#### Generic QOL instruments

The SF-36 was developed by a medical outcomes health survey. Broadly, it consisted of 8 dimensions: physical functioning, role limitations due to physical health problems, bodily pain, general mental health covering psychological distress and well-being, role limitations due to emotional problems, social functioning, vitality, and general health perceptions [38]. The internal consistency for the overall scale was 0.95 [38]. The test-retest correlations were more than 0.8 in the physical function and general health perceptions domains [39]. Correlations of convergent validity between the SF-36 and the WHOQOL-BREF were: the physical component summary of SF-36 with the physical domains of WHOQOL-BREF was 0.48; and the mental component summary of SF-36 with the whole WHOQOL-BREF scale ranged from 0.6-0.75 [40].

The WHOQOL-BREF was a brief version of the QOL instrument developed from the WHOQOL-100. It comprised 26 items covering physical, psychological and social health, and environmental domains as well as overall QOL and health [41]. The internal consistency ranged from 0.75-0.86 [40]. The test-retest reliability correlation ranged from 0.76-0.8 in an interval of 2-4 weeks [42]. The content validity was assessed by assessing the item-domain correlations (0.53-0.78) and the inter-domain correlation (0.51-0.64) [42]. By convergent validation with SF-36, the mental health domain had a high correlation of 0.75, and the lowest correlation in the physical functioning domain was 0.51 [40]. Factorial validity revealed 4 domains, and known-group validation differentiated the study population between sick and well individuals [43].

The QLI was designed to measure both the satisfaction and importance of various aspects of life, including the four domains of health and functioning, psychological/spiritual, social and economic, and family [44]. This scale consisted of 66 items to rate for satisfaction and importance of QOL. The internal consistency alpha ranged from 0.73-0.99 [44]. The test-retest reliability was tested in a 2-week interval, and ranged from 0.68-0.79 [45]. Content validity was assessed by using the Content Validity Index, with an acceptable rating level [46]. By convergent validation with the Life Satisfaction Scale, the correlation ranged from 0.61-0.93; factor analysis derived 4 domains [45].

The EQ-5D consisted of 6 items covering 5 dimensions of health: mobility, self-care, usual activities, pain/discomfort, and anxiety/depression, plus a global question to rate general health state [47]. The test-retest reliability was tested over a 1-week interval and reported as 0.86 for group level coefficients averaged over health states [48]. The content validity was verified by the research panel. Using convergent validation with the Hospital Anxiety and Depression Scale, the correlation was reported respectively as 0.44 (Anxiety scale) and 0.51 (Depression scale) [49].

#### Cancer-specific QOL instruments

The CARES-SF contained 59 items, covering physical, psychological, medical interaction, marital, and sexual domains [50]. In Schag et al.'s validation study, the reliability of internal consistency had an estimated alpha ranging from 0.61-0.85, and the test-retest correlation was 0.92 with a 1-month interval. The content validity of this scale was assessed by experts. Using convergent validation with the CARES, the correlation ranged from 0.67-0.85. Factorial validity revealed 6 domains, and known-group validation was able to distinguish between normative and rehabilitation individuals [50].

The EORTC QLQ-C30 consisted of 30 items and included 5 functional domain scales, such as physical, role, emotional and social functions, along with disease-specific symptoms, a financial impact domain, and two items related to global health status and QOL [51]. The internal consistency with an estimated alpha ranged from 0.74-0.86 [51]. The test-retest correlation over a 4-day interval ranged from 0.82-0.91 [52]. By convergent validation with the CARES, the correlation was respectively reported as 0.46 (Social domain), 0.56 (Psychological domain), 0.69 (Pain symptoms), and 0.71 (Physical domain) [53].

The FACT-G included 27 items and covered 4 primary QOL domains: physical, emotional, social and functional well-being [54]. Cella et al.'s validation report shows an internal consistency alpha of 0.89 for the total instrument, and a test-retest correlation ranging from 0.82-0.92 over a 3- to 7-day interval. The convergent validation with the Functional Living Index-Cancer Scale was 0.79. By using known-group validation, the FACT-G can significantly differentiate between patients at different stages of disease [54].

#### Cancer site-specific QOL instruments

The EORTC QLQ-Cx24 was developed to measure cervical cancer and its treatment-related issues. It covers the symptom experience, body image, and sexual/vaginal functioning subscales. The internal consistency of this scale ranged from 0.72-0.87 [55]. By convergent validation with the EORTC QLQ-C30, the correlation ranged from 0.4-0.48. The negative correlations of the body image subscale of QLQ-Cx24 with the emotional function and the global health/QOL of QLQ-C30 were minus 0.43 and 0.41. Known-group validation could distinguish subgroups of patients based on their clinical status [55].

The FACT-Cx consisted of 42 items: 27 items from the FACT-G plus 15 additional items to measure specific cervical cancer concerns. It was translated into 27 languages for use among a group of cross-cultural cancer patients [16]. The internal consistency alpha for each domain ranged from 0.69-0.89 [56]. Known-group validation could distinguish subgroups of patients with different types of treatment [56].

The QLICP-CE consisted of 40 items covering 5 domains of QOL: physical function, psychological function, social function, common symptoms and side-effects, and specific concerns of cervical cancer. Zhang et al. [36] reported that the internal consistency alpha for the overall scale was 0.68 and the test-retest reliability over a 3-day interval 0.95. The content validity was verified by experts. The factor loading of all items that remained in the scale was at least 0.6 by factor analysis [36].

#### QOL instruments for long-term cancer survivors

The CaSUN was developed using a needs-based approach to assess QOL among cancer survivors. This instrument consisted of 35 items covering 5 domains: information and medical issues, QOL, emotional and relationship issues, life perspective, and positive change issues [37]. The internal consistency had an estimated alpha of 0.96. Based on a 3-week interval, the test-retest correlation by an estimation of the Kappa coefficient was 0.13 [37]. The content validity was verified by the research panel and feedback from respondents. By convergent validation with the Hospital Anxiety and Depression Scale, the correlation was respectively reported as 0.4 (Anxiety subscale), and 0.34 (Depression subscale) [37].

### Summaries of psychometric properties

Additional file 3 also shows the psychometric properties of reliability and validity. The internal consistency of these 11 established multidimensional QOL instruments met the acceptable standards (0.68-0.99). In terms of the test-retest correlation, the average item-item correlation of CaSUN by Kappa coefficient was 0.13, below the acceptable level of correlation. Although the test-retest reliability of QLICP-CE was 0.95 by Pearson's coefficient test, the retest interval was a mere 2-3 days. Chawalow & Adesina [57] indicated that high test-retest correlation indices obtained over a short period (<1 week) may simply be reflected memory rather than actual stability of participants' perceptions. Consequently, higher test-retest correlations do not actually reflect the stability of an instrument if the retest interval is short.

For the establishment of validity, all these instruments had one or more types of construct validity reported. Most had conducted convergent validity which met acceptable standards. Other reported approaches of validity, such as factor analysis and known-group validation, were also considered acceptable.

## Discussion

There were 11 validated multidimensional QOL instruments, which could be classified into four categories: generic, cancer-specific, cancer site-specific and cancer survivor-specific instruments. All these instruments met the minimum requirements of reliability and validity, with the internal consistency of reliability varying from 0.68-0.99 and the test-retest reliability ranging from 0.6-0.95 based on the test of the Pearson coefficient. One or more types of validity supported the construct validity.

### General QOL measurement issues in cervical cancer survivors

The original versions of these 11 QOL instruments were mainly developed in Europe and North America, therefore how to select those that would be most appropriate for Chinese cervical cancer survivors requires careful consideration by researchers.

Among the generic scales, the WHOQOL-BREF was the most often-used scale among QOL studies in cervical cancer survivors. Some studies chose this scale because it had been translated and validated in their language [24,25]. Hence, these studies chose generic scales based on practical issues. One study chose the generic scale of QLI because there was a control group from the general population [26]. Generic scales were designed and validated in the general population. If the study objectives aimed at making a comparison of QOL between cancer survivors and the general population, choosing one of the generic scales would be suitable. However, while these generic instruments may be useful for making comparisons of QOL between cervical cancer survivors and the general population, they may not be sensitive enough to detect the impact of cancer and cancer treatment on QOL among cervical cancer survivors.

The majority of QOL studies in cervical cancer survivors chose cancer-specific scales. EORTC QLQ-C30 and FACT-G were the most frequently used. It is possible that cancer-specific scales are more responsive to changes than their generic counterparts, because cancer-specific instruments cover items in addressing the effects of cancer and related treatment on QOL. In consequence, it would be logical to speculate that cancer-specific scales would be more appropriate than generic scales in assessing QOL among cancer survivors. However, this speculation is only partially substantiated. Due to a failure to identify concerns specific to cervical cancer, these instruments may not be the most suitable for assessing QOL among cervical cancer survivors.

Cancer site-specific instruments may achieve greater specificity and sensitivity than either generic or cancer-specific scales, as site-specific scales cover general cancer-specific issues and address specific concerns related to cervical cancer. There were three site-specific instruments used by studies in our review: EORTC QLQ-Cx24, FACT-Cx and QLICP-CE. It may be speculated that these cancer site-specific scales are the most suitable choice for QOL studies in cervical cancer survivors. Yet these scales are more concerned with the immediate effects of cancer and acute cancer treatment, so that they are not appropriate for cervical cancer survivors due to the lack of items covering the long-term sequelae of cervical cancer, such as loss of fertility, sexual dysfunction, fear of recurrence, and body image disturbance [58].

In addition, cancer survivors reported positive changes in life outlook, self-growth, precious life, and an appreciation of their relationships with others [37]. All 11 QOL instruments used in cervical cancer survivors paid less attention to the positive outcome of the cancer survivorship experience. In more recent years, the trend of QOL instrument production has continued to emerge for cancer survivors, particularly for long-term (more than 5 years) cancer survivors with an emphasis on positive outcomes [10], such as the Quality of Life Scale for Adult Cancer Survivors (QLACS) by Avis et al. [59] and the Impact of Cancer (IOC) by Zebrack et al. [60]. However, these long-term cancer survivor-specific instruments have not been applied to the population of cervical cancer survivors.

According to Ashing-Giwa's contextual model [14], socio-ecological, cultural and healthcare system-related factors are essential components in the systemic level of QOL among cancer survivors. Additional file 2 shows the paradigm, domains, components and item distribution. These 11 multidimensional QOL instruments did not adequately incorporate the contextual milieu. In other words, there were few items that captured the contextual domain of QOL, such as socio-ecological and cultural issues. Even if the instrument of WHOQOL-BREF had an environmental domain, this scale covered too few items to measure the environmental domain of QOL adequately. Therefore, neither of these instruments was comprehensive enough to address or cover all QOL issues among cervical cancer survivors.

### Specific issues of QOL measurement in Chinese cervical cancer survivors

Although the instruments of EORTC QLQ-C30, FACT-G, and FACT-Cx had been applied to Chinese cervical cancer survivors, few studies calculated its reliability and validity when applied to Chinese women. Only one study by Wan et al. [61] established and reported FACT-G as having good reliability and validity among different types of Chinese cancer patients.

Recently, Zhang and colleagues developed the scale of QLICP-CE and validated it among a group of Chinese women with cervical cancer [36]. The domains and items included in the scale of QLICP-CE were mainly based on the instruments of EORTC QLQ-C30 and FACT-Cx. The QLICP-CE emphasized the aspects of women's appetite and sleep, as within Chinese culture good appetite, sleep and energy are highly regarded in daily life [62]. Due to Chinese communities viewing sex as a taboo topic, the QLICP-CE consists of just one item to measure the issues of sexual health. As sexuality is one of the essential components of QOL [63], this scale failed to address an important aspect of QOL for Chinese cervical cancer survivors.

Furthermore, culture is a major determinant of QOL, as perceptions of QOL are embedded in cultural beliefs about what constitutes normality and health [64]. At the individual level, the components of health practices, health utilization and perceived health practice should be measured in a culturally sensitive manner, because in Chinese culture the beliefs of Taoism (human beings should live in harmony with nature, that is, with 'Tao' as the way of life) and traditional Chinese medicine (TCM) (expanded from Taoism, it views health as harmony between vital energy - known as Qi - within and between the body and its environment) are dominate the views of health and health utilization [62,65]. These beliefs are different from those of Western people, therefore perceptions of QOL logically also differ between China and the West. Due to differences of social backgrounds and healthcare systems, at the systemic level the components of socio-economic status, access to and satisfaction with health care, and role and relationship changes should be particularly emphasized. Since the family relationship and kinship play very important roles in daily life in Chinese communities [62], roles and relationship changes due to cancer and treatment greatly influence Chinese women's QOL.

### Limitations of the review

In searching for literature, the 5 electronic databases used provided a comprehensive coverage of key English and Chinese medical, nursing and health-affiliated journals. However, the titles and abstracts were screened only by the first author. In order to compensate for this limitation, all articles were screened using a checklist based on clear inclusion and exclusion criteria. Additionally, all eligible articles were agreed upon by the research team. Other limitations include that the assessment of the psychometric properties of QOL instruments was limited to the reliability and validity. This review failed to assess other instrument properties, such as cross-cultural acceptability, responsiveness, and acceptability, because those properties were seldom reported by the instrument developers.

## Conclusion

According to this review, a total of 11 validated multidimensional instruments have been used to assess QOL among cervical cancer survivors. Almost all these QOL instruments were originally developed in North America or Europe. Due to cultural differences between these regions and China, further research needs to explore culturally specific issues in detail, such as what QOL domains are known to be important for Chinese women.

Regarding the issue of instrument selection, choosing an instrument for Chinese cervical cancer survivors should first take consideration of the QOL instruments' psychometric properties. Based on this review, all 11 instruments met the minimum requirements of reliability and validity. Secondly, instrument selection should be based on the purpose of investigation. From the previous discussion, if a study aims to compare the QOL of Chinese cervical cancer survivors with that of the general Chinese female population, the WHOQOL-BREF could be one of the potential instruments. By contrast, if the purpose of the study is to investigate QOL among survivors of different types of cancer including cervical cancer, QLACS, CaSUN and IOC should be translated and applied to Chinese cervical cancer survivors. Lastly, if the aim is simply to investigate QOL among short-term cervical cancer survivors, the QLICP-CE would be a potential choice.

Finally, instrument selection for Chinese cervical cancer survivors also needs to consider the comprehensiveness of the instruments. This issue could be addressed by incorporating different types of QOL instruments based on the purpose of investigation. However, choosing multiple QOL instruments, there is a high possibility that more time will be required of respondents. Consequently, further research is needed to develop an instrument tailored to assessing QOL for Chinese cervical cancer survivors across the whole survivorship, including immediately after diagnosis, in the short term (less than 5 years), and in the long term (more than 5 years).

## Competing interests

The authors declare that they have no competing interests.

## Authors' contributions

The first author was responsible for conducting the literature review and drafting the manuscript. All authors were involved in planning, reviewing, discussion, reporting, and approval of the final manuscript.

## Supplementary Material

Additional file 1 Categories of the established multidimensional QOL instruments adopted by studies in cervical cancer survivors. Click here for file

Additional file 2 The paradigm, domains, components and distribution of items across 11 multidimensional QOL instruments. Click here for file

Additional file 3 The psychometric properties of the 11 established multidimensional QOL instruments. Click here for file
