# Supplementary material for: Quality of life measurement in women with cervical cancer: implications for Chinese cervical cancer survivors
Source: Health Qual Life Outcomes. 2010 Mar 19;8:30. doi: 10.1186/1477-7525-8-30 (PMC2852383; doi:10.1186/1477-7525-8-30)
Supplement: Additional file 1 — Categories of the established multidimensional QOL instruments adopted by studies in cervical cancer survivors. [file 1477-7525-8-30-S1.DOC]

**Additional File 1**

**Categories of the established multidimensional QOL instruments adopted by studies in cervical cancer** survivors

|  | **Questionnaires** | **Origin** | **Levels and domains** | **Sample items** | **Reference** |
| --- | --- | --- | --- | --- | --- |
| Generic | MOS SF-36 | USA | *Individual level*: physical function, role-physical, bodily pain, general health, vitality, mental health, health change;  *Systemic level*: social functioning, role-emotional | *“In general, how would you say your health is?”; “During the past 4 weeks, have you been a happy person?”* | [www.sf36.com](http://www.sf36.com/) |
|  | WHOQOL-BREF | Europe | *Individual level*: physical, psychological, overall health  *Systemic level*: social, environmental conditions | *“How much do you enjoy your life?”; “Do you have enough energy for everyday life?””* | [www.who.int](http://www.who.int/) |
|  | QLI | USA | *Individual level:* physical, psychological and spiritual dimensions  *Systemic level:* interpersonal, economic and environmental dimensions | *“How satisfied are you with/how important is your health?”; “How satisfied are you with/how important is your faith in God?”* | [www.qolid.org](http://www.qolid.org/) |
|  | EQ-5D | Europe | *Individual level:* mobility, self-care, usual activities, pain/discomfort, anxiety/depression, overall health state | *“Mobility: 1. I have no problems walking about, 2. I have some problems in walking about, 3. I am confined to bed”* | [www.euroqol.org](http://www.euroqol.org/) |
| Cancer-specific | CARES-SF | USA | *Individual level:* physical, psychological and sexual domains  *Systemic level:* social, medical interaction, marital domains  *Mixed levels:* miscellaneous subscales and items | *“I have difficulties in activities of daily living”; “I have difficulty communicating with my partner”;* | [www.qolid.org](http://www.qolid.org/) |
|  | EORTC QLQ-C30 | Europe | *Individual level*: symptoms, physical function, cognitive functioning, emotional functioning, global QOL  *Systemic level*: role functioning, social functioning | *“Have you felt nauseated”; “Has your physical condition or mental treatment interfered with your social activities?”* | [www.eortc.be](http://www.eortc.be/) |
|  | FACT-G | USA | *Individual level*: physical well-being, emotional well-being, functional well-being  *Systemic level*: social well-being | *“I have nausea”; “I am bothered by the side-effects of treatment”* | [www.facit.org](http://www.facit.org/) |
| Cancer site-specific | EORTC QLQ-CX24 | Europe | *Individual level*: cervical cancer-specific symptoms and function | *“Have you had abnormal bleeding from your vagina?”; “Have you felt less feminine as a result of your disease or treatment?”* | [www.eortc.be/](http://www.eortc.be/) |
|  | FACT-CX | USA | FACT-G plus items of cervical cancer-specific concerns within the individual levels | *“My vagina feels too narrow or short”; “I have trouble controlling my urine”* | [www.facit.org](http://www.facit.org/) |
|  | QLICP-CE | China | *Individual level*: physical function, psychological function, common cancer-specific symptoms and side-effects, cervical cancer-specific concerns  *Systemic level*: social function | *“Do you have a good appetite?”; “Do you feel worried or depressed?”* | Zhang et al. (2009) |
| Survivor-  specific | CaSUN | Australia | *Individual level*: existential survivorship, quality of life, information;  *Systemic level*: comprehensive cancer care, relationships | *“I need to help to manage ongoing side-effects and/or complications of treatment”; “I need local healthcare services that are available when I require them…”* | Hodgkinson et al. (2007) |

SF-36(Medical Outcome Study questionnaire-Short Form-36items), WHOQOL-BREF(World Health Organization Quality of Life–Brief), QLI (Quality of Life Index), EQ-5D (European Quality of Life Scale-5 dimensions), CARES-SF(Cancer Rehabilitation Evaluation System-Short Form), EORTC QLQ-C30(European Organization for Research Treatment’s Quality of Life Questionnaire), FACT-G(Functional Assessment of Cancer Therapy-General), EORTC QLQ-Cx24(Quality of Life Questionnaire-Cervix-24items), FACT-Cx(Functional Assessment of Cancer Therapy-Cervix), QLICP-CE (Quality of Life Instruments for Cancer Patients-Cervical Cancer), CaSUN (Cancer Survivors’ Unmet Needs)
