# Supplementary material for: Quality of life measurement in women with cervical cancer: implications for Chinese cervical cancer survivors
Source: Health Qual Life Outcomes. 2010 Mar 19;8:30. doi: 10.1186/1477-7525-8-30 (PMC2852383; doi:10.1186/1477-7525-8-30)
Supplement: Additional file 2 — The paradigm, domains, components and distribution of items across 11 multidimensional QOL instruments. [file 1477-7525-8-30-S2.DOC]

**Additional File 2**

**The paradigm, domains, components and distribution of items across 11 multidimensional QOL in**struments

| **Paradigms** | **Domains**↓ | QOL Instruments → | **SF-36** | **WHOQOL-BREF** | **QLI** | **EQ-5D** | **CARES-SF** | **EORTC QLQ**  **C30** | **FACT-G** | **EORTC QLQ**  **-Cx24** | **FACT-Cx24** | **QLICP-CE** | **CaSUN** |
| --- | --- | --- | --- | --- | --- | --- | --- | --- | --- | --- | --- | --- | --- |
|  |  | Category → | Generic | | | | Cancer-specific | | | Cancer site-specific | | | Survivor  -specific |
|  |  | Total # Items →  Components from contextual model ↓ | 36 | 26 | 66 | 6 | 59 | 30 | 27 | 24 | 42 | 40 | 35 |
|  |  |  | Item distribution among components↓ | | | | | | | | | | |
| **Individual Level** | General  Health | Health status | 19 | 9 | 16 | 1 | 11 | 7 | 7 |  | 9 | 8 | 4 |
|  |  | Co-morbidity | 2 |  |  | 1 | 3 | 5 |  | 20 | 11 | 11 | 1 |
|  | Medical Factors | Age at diagnosis |  |  |  |  |  |  |  |  |  |  |  |
|  |  | Cancer characteristics |  |  |  |  | 5 | 6 |  |  |  |  |  |
|  | Health Efficacy | Health practices |  |  |  | 1 |  |  |  |  |  |  |  |
|  |  | Health utilization |  |  |  | 1 |  |  |  |  |  |  |  |
|  |  | Perceived  health efficacy |  |  |  | 1 |  |  |  |  |  |  |  |
|  |  | Medical adherence |  |  |  |  | 1 |  |  |  |  |  |  |
|  | Psycho-logical  Health | Emotional distress | 3 | 3 | 14 | 1 | 12 | 5 | 6 | 4 | 6 | 12 | 3 |
|  |  | Cognitive function | 5 | 1 |  |  | 1 | 2 |  |  |  | 2 |  |
|  |  | Positive psychological feelings | 2 | 1 | 2 |  |  |  | 5 |  | 7 |  |  |
| **Systemic Level** | Socio-ecological | Socio-economic status |  | 6 | 16 |  | 6 | 1 | 2 |  | 2 | 2 | 5 |
|  |  | Life burden |  | 1 |  |  |  |  |  |  |  |  | 3 |
|  |  | Social support | 2 | 1 | 4 |  | 6 |  | 2 |  | 2 | 2 | 5 |
|  |  | Roles/relation-ship changes | 3 | 2 | 8 |  | 5 | 4 | 5 |  | 5 | 1 | 4 |
|  | Cultural | Spirituality |  | 1 | 4 |  |  |  |  |  |  | 1 | 2 |
|  |  | Acculturation |  |  |  |  |  |  |  |  |  |  |  |
|  |  | Interconnected-ness |  |  |  |  |  |  |  |  |  |  |  |
|  | Healthcare System | Access to healthcare |  |  | 2 |  | 1 |  |  |  |  | 1 | 7 |
|  |  | Satisfaction with healthcare |  | 1 |  |  | 3 |  |  |  |  |  |  |
|  | Demo-  graphic | Chronological age |  |  |  |  |  |  |  |  |  |  |  |
| Unclear categories of items | | |  |  |  |  | 5 |  |  |  |  |  | 1 |
