# Supplementary material for: Quality of life measurement in women with cervical cancer: implications for Chinese cervical cancer survivors
Source: Health Qual Life Outcomes. 2010 Mar 19;8:30. doi: 10.1186/1477-7525-8-30 (PMC2852383; doi:10.1186/1477-7525-8-30)
Supplement: Additional file 3 — The psychometric properties of the 11 established multidimensional QOL instruments. [file 1477-7525-8-30-S3.DOC]

**Additional File 3**

**The psychometric properties of the 11 established multidimensional QOL instruments**

| **Instruments** | **Reliability** | | **Content Validity** | **Construct Validity** | |
| --- | --- | --- | --- | --- | --- |
|  | Internal consistency (Cronbach’s α) | Test-retest correlations |  | Convergent validity | Other approaches |
| SF-36 | 0.95 | r=0.6 in social function, r>0.8 in physical function, vitality and general health perceptions (2-week interval) |  | Convergent validation with WHOQOL-BREF, r=0.48 (Physical component summary vs. Physical domain); r=0.60-0.75 (Mental component summary vs. 4 domains of WHOQOL-BREF) |  |
| WHOQOL-BREF | 0.75-0.86 | r=0.76-0.8 (2- to 4-week interval) | Assessed by item-domain correlations (0.53-0.78) and inter-domain correlations (0.51-0.64) | Convergent validation with SF-36, r=0.51 (Physical functioning); r=0.75 (Mental health); r=0.54 (Social functioning) | Factor analysis revealed 4 factors (eigenvalues >1.0);  Known-group validation differentiates sick and well individuals |
| QLI | 0.73-0.99 | r=0.68-0.79 (2-week interval) | Assessed using the Content Validity Index with an acceptably high rating level | Convergent validation with the Life Satisfaction Scale, r=0.61-0.93 | Factor analysis derived 4 domains |
| EQ-5D |  | r=0.86 (7-day interval) | Feedback from respondents | Convergent validation with HADS, r=0.44 (Anxiety subscale)  r=0.51 (Depression subscale) |  |
| CARES-SF | 0.85 | r=0.92 (1 month interval) | Selected from the CARES by experts | Convergent validation with CARES, r=0.67-0.85 | Factor analysis derived for 6 domains; Known-group distinguishing between normative and rehabilitation samples |
| EORTC QLQ-C30 (Version 3) | 0.74-0.86 | r=0.82-0.91 (4-day interval) | Verified by cancer patients’ feedback and the research team | Convergent validation with CARES, r=0.71 (Physical domain); r=0.56 (Emotional domain); r=0.46 (Social domain);  r=0.69 (Pain domain) |  |
| FACT-G (Version 4) | 0.89 | r=0.82-0.92 (3- to 7-day interval) |  | Convergent validation with the Function Living Index-Cancer scale, r=0.79 | Using Known-group significantly differing between patients in different stages of their disease |
| EORTC QLQ-Cx24 (Phase 3 module) | 0.72-0.87 |  |  | Convergent validation with EORTC QLQ-C30, r=0.4-0.48 (Symptom experience vs. Functioning subscales); r=-0.43 (Body image vs. Emotional function subscale); r=-0.41 (Body image vs. Global health/QOL) | Known-group validation differing between? subgroups of patients in terms of treatment status |
| FACT-Cx (Version 4) | 0.69-0.89 |  |  |  | Known-group validation differing between? subgroups of patients with different types of treatment |

| QLICP-CE (Version 1) | 0.68 | r=0.95 (2- to 3-day interval) | Verified by the research panel and experts |  | Factor analysis derived for 12 domains, items with eigenvalues ≥0.6 retained |
| --- | --- | --- | --- | --- | --- |
| CaSUN  (Version 1) | 0.96 | Average item-item Kappa coefficient=0.13 (3-week interval) | Verified by research panel and feedback from respondents | Convergent validation with HADS,  r=0.4 (Anxiety subscale); r=0.34 (Depression subscale) | Factor analysis revealed 5 domains |

SF-36(Medical Outcome Study questionnaire-Short Form-36items), WHOQOL-BREF(World Health Organization Quality of Life–Brief), QLI (Quality of Life Index), EQ-5D (European Quality of Life Scale-5 dimensions), CARES-SF(Cancer Rehabilitation Evaluation System-Short Form), EORTC QLQ-C30(European Organization for Research Treatment’s Quality of Life Questionnaire), FACT-G(Functional Assessment of Cancer Therapy-General), EORTC QLQ-Cx24(Quality of Life Questionnaire-Cervix-24items), FACT-Cx(Functional Assessment of Cancer Therapy-Cervix), QLICP-CE (Quality of Life Instruments for Cancer Patients-Cervical Cancer), CaSUN (Cancer Survivors’ Unmet Needs.
